# Impact of Lifestyle Intervention Programs for Children and Adolescents with Overweight or Obesity on Body Weight and Selected Cardiometabolic Factors—A Systematic Review

**DOI:** 10.3390/ijerph18042061

**Published:** 2021-02-20

**Authors:** Beata Bondyra-Wiśniewska, Joanna Myszkowska-Ryciak, Anna Harton

**Affiliations:** Department of Dietetics, Institute of Human Nutrition Sciences, Warsaw University of Life Sciences (WULS), 159C Nowoursynowska Str, 02-776 Warsaw, Poland; joanna_myszkowska_ryciak@sggw.edu.pl

**Keywords:** childhood obesity, intervention program, diet, lifestyle, BMI, excess body weight, dyslipidemia, blood pressure

## Abstract

Excessive body mass is a health problem among children and adolescents that contributes to the occurrence of lipid disorders and abnormal blood pressure. Effective treatment of excessive body mass in children is essential for the health of population in the future. The aim of the study was to identify universal components of lifestyle interventions in children and adolescents with overweight or obesity leading to weight loss and improvement of selected cardiometabolic parameters. The review included studies from the PubMed and Google Scholar databases published in 2010–2019, which were analyzed for eligibility criteria including age of the participants, BMI defined as overweight or obese, nutritional intervention and the assessment of BMI and/or BMI z-score and at least one lipid profile parameter. Eighteen studies were included in the review, presenting the results of 23 intervention programs in which a total of 1587 children and adolescents participated. All interventions, except one, were multi-component. Data analysis suggests a relationship between a decrease in BMI and/or BMI z-score with diet and physical activity, the involvement of a dietician/nutrition specialist and physician in the treatment team and a longer duration of intervention. Moreover, it seems that a decrease in BMI is mostly associated with decreases in total cholesterol, triglycerides, low density lipoprotein cholesterol and blood pressure. No change in BMI and/or BMI z-score is associated with no change in blood pressure. Our data can be used by public health authorities to design effective weight loss programs for children and adolescents.

## 1. Introduction

Steadily increasing prevalence of excess body weight among children and adolescents is currently one of the greatest challenges for public health authorities worldwide. By 1997, obesity was officially recognized by experts of the World Health Organization (WHO) as a global epidemic among children, adolescents and adults [1]. According to the WHO data, in 2016, 41 million children under the age of 5 and 340 million children/adolescents aged 5–19 were overweight and obese worldwide. The number of children and adolescents with obesity worldwide has increased from 11 million in 1975 to 124 million in 2016 [2]. The Organization for Economic Cooperation and Development (OECD) reports that excess body weight occurs on average in 25% of children and adolescents aged 2–19 [3]. In the last four decades, there has also been an increase of the global age-standardized mean body mass index (BMI) of children and adolescents aged 5–19 from 17.2 to 18.6 kg/m^2^ in girls (increase of 0.32 kg/m^2^ in one decade) and from 16.8 to 18.5 kg/m^2^ in boys (increase of 0.4 kg/m^2^ in one decade) [4].

Childhood obesity is conducive to the occurrence of many disorders resulting from excess body weight, such as lipid disorders, abnormal blood pressure, increased glucose levels, insulin resistance and exercise intolerance. They lead to the development of many chronic diseases in adulthood, especially with the persistently overweight, including cardiovascular diseases, hypertension, type 2 diabetes and metabolic syndrome [3,5,6,7]. The continuous worsening of the problem of obesity among children and adolescents causes not only an increase in the incidence of diseases, but also a shift in the time of their onset in younger age groups [8]. Due to the large number of childhood obesity disorders, this review focuses on cardiometabolic factors.

Excess body weight is the most common cause of dyslipidemia in children and adolescents [9]. Researchers from Mexico observed an approximately 2–3 times higher risk of dyslipidemia in children and adolescents with overweight or obesity aged 2–16 years compared to their peers with normal body weight [10,11]. Data from Denmark showed an over 6-fold higher risk of dyslipidemia in children with obesity aged 6–19 [12]. Polish studies conducted among 778 adolescents aged 16–18 showed that high BMI values correlated with a greater frequency of high triglyceride (TG) levels (odds ratio (OR) = 1.7) and low levels of high-density lipoprotein cholesterol (HDL-C) (OR = 3.5) [13]. Iranian researchers made similar observations among children aged 7–12 [14]. They showed that children with abdominal obesity had higher risk of dyslipidemia compared to their peers with normal body weight. High levels of TG and low-density lipoprotein cholesterol (LDL-C) occurred in these children 6 times more often and high levels of total cholesterol (TC) almost 3 times more often. Data from the Bogalusa Heart Study conducted in the United States indicated that school-age children with excess body weight had more than 2 times greater risk of elevated levels of TC. For other lipid parameters, the risk of lipid disorders was even greater—for LDL-C it was 3.0, for HDL-C—3.4 and for triglycerides—7.1 [15].

According to various sources, the overall incidence of dyslipidemia in children and adolescents with overweight or obesity ranges from about 17% to almost 74% (Table 1). The prevalence of dyslipidemia in children and adolescents with overweight or obesity as compared to their normal-weight peers varies by age group and country. In Mexico, dyslipidemia occurred in 42–49% of children and adolescents with normal body weight and 62–74% with obesity [10,11], while in Denmark these values were 5.2% and 28.0%, respectively [12]. In most of the studied groups, the most common lipid disorder was an increased triglyceride level, the frequency of which was greater in children with obesity than with overweight.

Unhealthy eating patterns and sedentary behavior can be passed on to the next generation. This contributes to the increased risk of childhood obesity [16]. In Germany, it has been observed that the proportion of obese children and adolescents entering lifestyle intervention programs has increased over the decade. Hypertension and dyslipidemia were similarly more frequent [17]. Dealing with the problem of excess body weight in childhood is important in the context of health of the population in the future. Therefore, it is so important to apply an effective intervention promoting proper health behavior as early as possible.

In the available literature, there is still insufficient data showing the impact of the intervention on changes in body weight and cardiometabolic parameters. This article attempts to answer the question which components of lifestyle intervention programs in children and adolescents with excess body weight contribute to success in the context of weight loss and an improvement of cardiometabolic parameters (such as lipid parameters and blood pressure).

**Table 1:** Prevalence of dyslipidemia in children and adolescents with normal weight, overweight and obesity (both sexes).

Country, Year [Reference]	Age (Years)	*n*	Criteria for Measuring Body Weight		Frequency of Dyslipidemia (%)					Study Design	Odds Ratio for Dyslipidemia
					Dyslipidemia	High TG	High TC	High LDL-C	Low HDL-C		
Argentina, 2014 [18]	8–14	139	overweight and obesity	BMI z-score for overweight: 1–2; BMI z-score for obesity > 2	50.4	31.9	11.9	10.7	29.7	nd	nd
Brazil, 2012 [19]	6–10	147	overweight and obesity	BMI ≥ 85th pc	28.0	10.2	11.6	8.8	8.8	cross-sectional study	nd
Brazil, 2012 [20]	7–14	698	normal	BMI ≥ 3rd and < 85th pc	24.0	nd	nd	nd	nd	cross-sectional study	1.0 (ref.)
		116	overweight and obesity	BMI ≥ 85th pc	26.4	nd	nd	nd	nd		3.4 (*p* < 0.001)
Brazil, 2009 [21]	2–19	383	no excess weight	BMI < 85th pc	nd	4.7	47.8	36.8	6.0	cross-sectional study	nd
		48	overweight	BMI ≥ 85th and < 95th pc	nd	14.6	45.8	50.0	4.2		
		63	obesity	BMI ≥ 95th pc	nd	27.0	74.6	55.6	7.9		
China, 2016 [22]	6–18	1649	normal	BMI criteria according to WGOC	nd	11.4	10.9	4.8	3.1	cross-sectional study	nd
			overweight		nd	18.5	10.0	6.2	12.3		
			obesity		nd	37.2	12.1	9.9	13.9		
Denmark, 2017 [12]	6–19	1639	normal	BMI: 10th—90th pc	5.2	0.8	2.8	2.0	1.3	population-based cohort study	nd
		1421	overweight and obesity	BMI > 90th pc	28.0	14.8	7.1	6.8	12.7		
India, 2017 [23]	5–18	65	obesity	BMI ≥ 95th pc	63.0	46.2	40.0	60.0	40.0	cross-sectional study	nd
Iran, 2015 [14]	7–12	100	control	BMI < 85th pc and WC < 90th pc	nd	13.0	13.0	5.0	11.0	case control study	nd
		100	overweight	BMI: 85th -95th pc and WC < 90th pc	nd	20.0	8.0	8.0	13.0		
		100	general obesity with central obesity	BMI ≥ 95th pc and WC ≥ 90th pc	nd	49.0	28.0	24.0	38.0		
Iran, 2015 [24]	11–18	2231	normal	nd	nd	14.7	6.2	3.6	25.5	cross-sectional study	nd
		412	overweight and obesity		nd	10.5	5.3	2.9	20.1		
Iran, 2011 [25]	6–18	2064	overweight and obesity	BMI ≥ 85th pc	69.9	49.9	32.4	23.0	24.4	retrospective study	nd
Iran, 2009 [26]	4–18	50	normal	BMI: 50th–85th pc	nd	nd	nd	nd	nd	case control study	nd
		72	overweight	BMI z-score: 1–2	26.2	nd	nd	nd	nd		
		41	moderately obesity	BMI z-score: 2–2.5	16.9	nd	nd	nd	nd		
		117	severely obesity	BMI z-score > 2.5	56.8	nd	nd	nd	nd		
Mexico, 2016 [10]	11–16	193	normal	BMI criteria according to IOTF	42.0	nd	nd	nd	nd	population-based cross-sectional nutritional survey	1.0 (ref.)
		58	overweight		60.0	nd	nd	nd	nd		2.07 (*p* < 0.05)
		42	obesity		62.0	nd	nd	nd	nd		2.21 (*p* < 0.05)
Mexico, 2015 [11]	2–10	241	normal	BMI criteria according to IOTF	49.4	32.8	14.9	12.0	19.5	population-based cross-sectional nutritional survey	1.0 (ref.)
		47	overweight		63.8	48.9	19.1	8.5	36.2		1.76
		49	obesity		73.5	59.2	18.4	16.3	55.1		2.79
Pakistan, 2019 [27]	10–16	58	overweight	BMI ≥ 85th and < 95th pc	29.3	20.7	0.0	0.0	15.5	cross-sectional study	nd
		41	obesity	BMI ≥ 95th pc	61.0	46.3	4.9	2.4	43.9		
Poland, 2011 [13]	16–18	69	overweight	BMI criteria according to IOTF	nd	36.2	nd	nd	21.7	nd	nd
		19	obesity		nd	63.2	nd	nd	26.3		
Poland, 2010 [28]	<10	91	obesity	BMI > 97th pc	25.0	14.1	17.2	48.8	22.0	nd	nd
Turkey, 2015 [29]	2–19	823	obesity	BMI ≥ 95th pc	42.9	21.7	18.6	13.4	19.7	retrospective study	nd
United Arab Emirates, 2018 [30]	4–19	216	overweight and obesity	BMI ≥ 85th pc	55.3	28.6	11.7	32.7	18.0	cross-sectional study	nd
United States, 2015 [31]	6–19	19,151	normal	BMI ≥ 5th and < 85th pc	13.8	nd	6.3	nd	6.8	cross-sectional study	nd
			overweight	BMI ≥ 85th and < 95th pc	22.3	nd	6.9	nd	14.8		
			obesity	BMI ≥ 95th pc	43.3	nd	11.6	nd	33.2		
United States, 2010 [32]	12–19	2008	normal	BMI > 5th and < 85th pc	14.2	5.9	nd	5.8	4.3	cross-sectional study	1.0 (ref.)
		514	overweight	BMI ≥ 85th and < 95th pc	22.3	13.8	nd	8.4	8.3		1.6
		603	obesity	BMI ≥ 95th pc	42.9	24.1	nd	14.2	20.5		3.0

BMI—body mass index; dyslipidemia—≥ 1 lipid abnormality; HDL-C—high-density lipoprotein cholesterol; IOTF—International Obesity Task Force; LDL-C—low-density lipoprotein cholesterol; nd—no data; pc—percentile; ref.—reference; TC—total cholesterol; TG—triglycerides; WC—waist circumference; WGOC—Working Group for Obesity in China.

### 1.1. Search Strategy

This study is a review of the literature on the effects of lifestyle interventions among children and adolescents with overweight or obesity. The aim was to identify common components of lifestyle interventions leading to weight loss and improvement of selected cardiometabolic parameters (such as lipid parameters and blood pressure). Electronic databases searched for this literature review included PubMed and Google Scholar. The databases were searched for articles published from 2010 to 2019 using controlled terms (MeSH) including: “Body Mass Index”, “children”, “adolescents”, “overweight”, “obesity”, “diet modification”. The search was carried out in January and February 2020. The references in these and relevant review articles were reviewed for additional articles that may have been overlooked during database searches. Only studies published in English were selected.

### 1.2. Eligibility Criteria

The study inclusion criteria were: (1) mean age between 5 and 18 years, (2) only participants with BMI defined as overweight or obesity, (3) any nutritional intervention in the treatment of overweight or obesity, (4) BMI or BMI z-score and (5) at least one lipid profile component (such as total cholesterol, low density lipoprotein cholesterol, high density lipoprotein cholesterol and triglycerides). Relevant secondary outcomes, if measured, were also collected. They included body composition assessment (lean mass, body fat, free-fat mass), waist circumference, waist to hip ratio and blood pressure. Only the full version articles were evaluated.

Studies have been excluded for the following reasons: (1) mean age below 4 and above 18 years, (2) participants with BMI defined as underweight or normal body weight, (3) nutritional program for the prevention of overweight or obesity, (4) no nutritional intervention, (5) the use of medicines or supplements that can change any outcome measure, (6) meta-analyses and reviews. No restriction was placed on geographical location.

**Figure 1:**
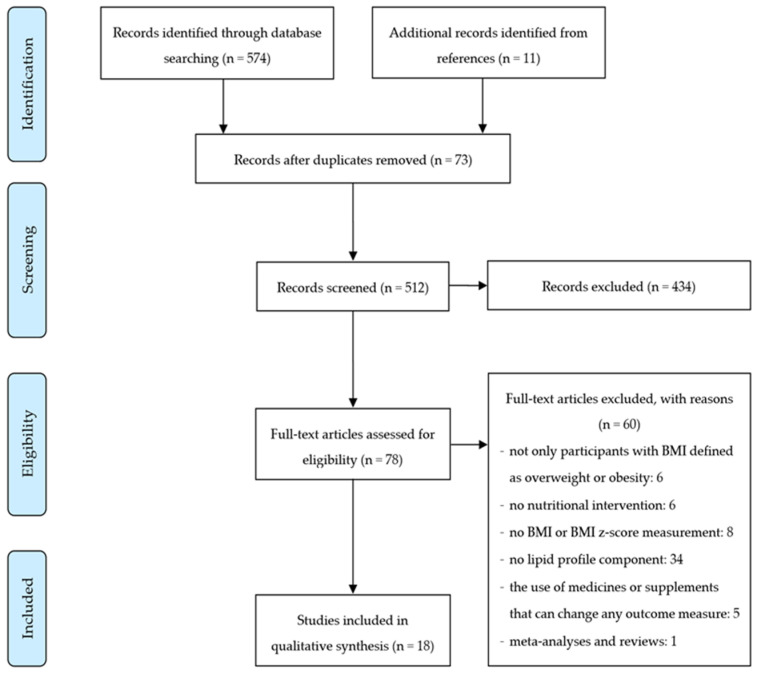
The flow diagram of the literature review process.

Figure 1 shows the flow diagram of the literature review process. Electronic searches identified 574 titles, of which 62 duplicate studies were removed. 512 titles and summaries were reviewed, of which 434 did not meet the eligibility criteria. At this stage, the most common reason for exclusion was the lack of nutritional intervention. The remaining 78 abstracts qualified for the full-text review. Of these, 60 did not meet the inclusion criteria, the most common reason being the lack of evaluation of lipid profile parameters. Finally, 18 studies eligible for review were identified presented the results of 23 nutritional interventions.

### 1.3. Statistical Analysis

All statistical analyses were conducted using Statistica version 13.1 (Copyright^©^StatSoft, Inc, 1984–2014, Cracow, Poland). For all tests, *p* < 0.05 was considered as significant.

The analyzed factors included the type of nutritional intervention, physical activity, parental and therapeutic team involvement, and the duration of the intervention. The duration of the intervention in the analyzed studies varied. Therefore, for the purposes of data analysis, two categories were distinguished: less than 6 months and a minimum of 6 months.

Correspondence analysis was used in the review—a multidimensional method to evaluate the relationship between two or more sets of variables [33,34]. The correspondence analysis was used to assess the relationship between components of lifestyle intervention programs and changes in BMI and/or BMI z-score as well as selected cardiometabolic parameters. The results of the analysis are presented in the form of a two-dimensional Burt matrix to better illustrate relationships between variables.

Depending on the assessed parameters, the analyses included a different number of intervention programs, which resulted from the availability of data. Intervention programs in which the parameter was not assessed were excluded from the analyses.

## 2. Results

### 2.1. Studies Characteristic

Data from the included studies were extracted and summarized in Table 2. The collected details included study characteristics: age, the number of participants, body weight evaluation criterion, duration, therapeutic team, type and effect of the intervention used. The studies were conducted in 7 European countries (9 studies), 3 countries in Asia (4 studies) and the United States (5 studies). All interventions were multi-component and included diet, nutritional behavioral component, physical activity, and/or parental involvement. Children and adolescents aged 6–18 were enabled in this study. A total of 1587 children and adolescents took part in all analyzed studies.

To classify excess body weight, different BMI diagnostic criteria were used in the analyzed studies. The most commonly used cut-off point was BMI ≥ 95th percentile and BMI ≥ 97–98th percentile. In two studies a reference to the International Obesity Task Force (IOTF) cut-offs as well as a BMI ≥ 85th percentile were used. In several studies a country-specific criteria, raw BMI and BMI z-score ≥ 2 were used. Two studies did not identify which references were used to define excess body weight.

Children and adolescents of both sexes participated in each study. Boys ranged from 8.3% to 65.4% of the study group, and in most studies the number of boys was in the range of 40–60%. In two research groups, it was not specified how many boys and girls were included at the beginning of the intervention.

In the majority of interventions there was no control group. In one case the results were compared to the values obtained in the group of healthy teenagers. In three, the impact of the intervention was compared to the results from standard care groups, and in one, the impact of a low-glycemic index diet was compared to a reduced-calorie diet (low fat, high fiber diet). Only in three groups the parameters were reassessed several weeks after the end of the intervention.

### 2.2. Nutritional and Physical Activity Interventions

#### 2.2.1. Nutritional Interventions

Nutritional interventions were heterogeneous and consisted of many components designed to improve diet quality and energy intake. The specific energy value of the diet was determined in 13 of 23 analyzed nutritional interventions (hereinafter referred to as diet) [35,37,39,40,41,42,43,46,47,48,49], of which 7 were caloric restriction [35,37,41,43,46,48]. Other groups used a normocaloric diet adapted to the current energy body needs. Most of these interventions also specified the percentage of individual macronutrients in the diet, including 12–25% protein, 15–35% fat, and 45–60% carbohydrates [37,39,40,41,42,43,46,47,48,49]. The effectiveness of various types of diets was assessed, including diets with a high and low glycemic index, plant-based with no added fat diet, a diet based on the American Heart Association (AHA) guidelines or based on the principles of the DASH diet. In one case, a ketogenic diet was used. In two studies, children took part in a weight loss camp, during which they received meals composed in accordance with national nutrition guidelines.

The most commonly used treatment for obesity (21 interventions) in children and adolescents was the intervention that used the nutritional behavioral component (hereinafter referred to as behavioral methods) [36,38,39,40,41,42,43,44,45,46,47,48,49,50,51,52]. Among them, a method such as self-monitoring has emerged (7 interventions) [36,43,47,49,50]. This method was based on keeping a food diary enabling the daily tracking of eating behavior [53]. Another method used was to set achievable nutritional goals such as eating breakfast, increasing intake of vegetables, fruits, low-fat dairy products, fish and whole grain products, limiting sweets and sweetened drinks (8 interventions) [39,42,44,45,46,50,51]. In 5 interventions, participants received cash or material rewards for achieving the set goals [36,49,50,51]. In 14 interventions, health education lessons were conducted, during which topics related to proper nutrition were discussed, including learning to read food labels, healthy cooking methods and healthy food choices [36,38,40,42,44,45,46,47,48,49,51,52].

#### 2.2.2. Physical Activity Interventions

Another important element used in the therapy of excess body weight was the implementation of physical activity. For most interventions, participants received a detailed guidance on the type and duration of physical activity (13 interventions, 8 of which were supervised) [35,36,37,38,39,40,44,46,48,50,51,52]. In the next 5 interventions, children were given general guidelines to exercise at least 30–60 min per day and were encouraged to limit their sedentary time [41,42,45,47]. In 2 interventions, participants additionally kept a physical activity diary or a physical activity questionnaire [44,47], and 3 interventions used physical activity monitoring with a physical activity tracker [36,44]. No information on physical activity was recorded in 5 interventions [36,43,49].

#### 2.2.3. The Impact of Nutritional and Physical Activity Interventions on the BMI and/or BMI z-Score

Eighteen interventions showed a significant decrease in BMI and/or BMI z-score, of which 15 groups were compared with baseline, and 3 with the control group [35,37,38,39,40,41,42,43,44,45,46,47,48,49,51,52]. In 5 groups there was no significant influence of the intervention on BMI and/or BMI z-score [36,44,49,50]. A statistically significant decrease in BMI and/or BMI z-score was noted in 12 of 13 interventions with a diet [35,37,39,40,41,42,43,46,47,48]. A significant decrease in BMI and/or BMI Z-score was observed in almost all interventions where the percentage of individual macronutrients in the diet was determined (10 interventions) [37,39,40,41,42,43,46,47,48]. Out of interventions that used the behavioral methods, 14 showed a significant reduction in BMI and/or BMI z-score compared to baseline [39,40,41,42,43,44,45,46,47,48,49,51] and in 2 interventions compared to the control group [38,52]. In the next 3 interventions there were no changes compared to the baseline [44,49,50] and in 2 intervention there were no change compared to the control group [36].

In 18 studies, intervention participants received recommendations to increase physical activity, of which 12 groups had a decrease in BMI and/or BMI z-score compared to baseline, and in another 3 compared to the control group. Overall, 15 interventions with recommendations for increased physical activity resulted in significant weight loss [35,37,38,39,40,41,42,44,45,46,47,48,51,52]. Similar observations were made in 3 out of 5 groups in which no recommendations for physical activity were given [43,49]. In the remaining study groups, BMI and/or BMI z-score did not change significantly.

**Figure 2:**
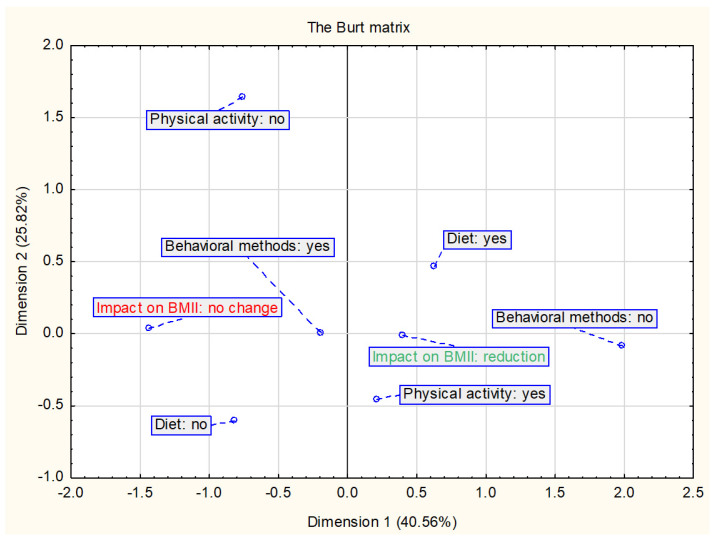
Presentation of the correspondence analysis results defining the relationship between the nutritional and physical activity interventions and changes in BMI and/or BMI z-score (*n* = 23).

The Burt matrix (Figure 2) shows the relationship between the nutritional and physical activity interventions and changes in BMI and/or BMI z-score. The analysis included all interventions assessed in this study (*n* = 23). The results suggest that a decrease in BMI and/or BMI z-score is most related to diet and physical activity interventions. In the studies assessed, physical activity has always been combined with nutritional intervention and is therefore also included in this analysis together.

#### 2.2.4. The Impact of Nutritional and Physical Activity Interventions on Selected Cardiometabolic Parameters

In the 14 interventions included in the review, the researchers noted significant improvements in at least one lipid profile parameter [35,36,37,39,40,42,44,45,46,48,49,50,51,52], of which 10 interventions decreased LDL-C [35,37,39,44,45,46,48,49,50,51], 9 decreased TC [37,39,45,46,48,49,50,51,52], 5 decreased TG [37,39,42,46,51], and 5 increased HDL-C [35,36,40,42,48]. A significant improvement in these parameters was noted in 7 diet interventions [35,37,39,40,42,46,48] and in 12 that used the behavioral methods [36,39,40,42,44,45,46,48,49,50,51,52]. In most of the interventions that significantly improved at least one parameter of the lipid profile, children received recommendations to increase physical activity (12 interventions), of which 11 groups received detailed guidelines on the type and duration of physical activity [35,37,39,40,42,44,45,46,48,50,51,52].

In 9 out 23 nutritional interventions, the researchers observed a significant reduction in systolic blood pressure (SBP) [36,37,38,39,40,43,46,49]. Six interventions used a diet [37,39,40,43,46] and 8 used behavioral methods [36,38,39,40,43,46,49]. Both were used in 5 studies. Almost half of the interventions did not significantly affect SBP (11 interventions, 6 of which used both diet and behavioral methods) [36,41,42,44,47,48,49,50,52]. Similar observations were made for diastolic blood pressure (DBP): 7 interventions showed a significant reduction in DBP [36,37,39,40,43,44], 5 of which used a diet [37,39,40,43] and 6 used behavioral methods [36,39,40,43,44]. Both were used in 4 interventions. More than half of the interventions (13 of 23) did not significantly affect DBP [35,36,41,42,44,46,47,48,49,50,52], of which 7 used both diet and behavioral methods [41,42,46,47,48,49].

Of the studies where participants were instructed to exercise, a reduction in SBP was reported in 1/3 (6 out of 18) [36,37,38,39,40,46], and half of the interventions had no significant effect [41,42,44,47,48,50,52]. Similarly, in the case of DBP. In 5 of the 18 interventions where recommendations for physical activity were provided, DBP values were significantly reduced [36,37,39,40,44], but 10 of the interventions did not have a significant effect [35,41,42,44,46,47,48,50,52]. In the remaining 3 interventions, there was no information about the effect on DBP [38,45,51].

#### 2.2.5. The Impact of Changes in BMI and/or BMI z-score on Selected Cardiometabolic Parameters

**Figure 3:**
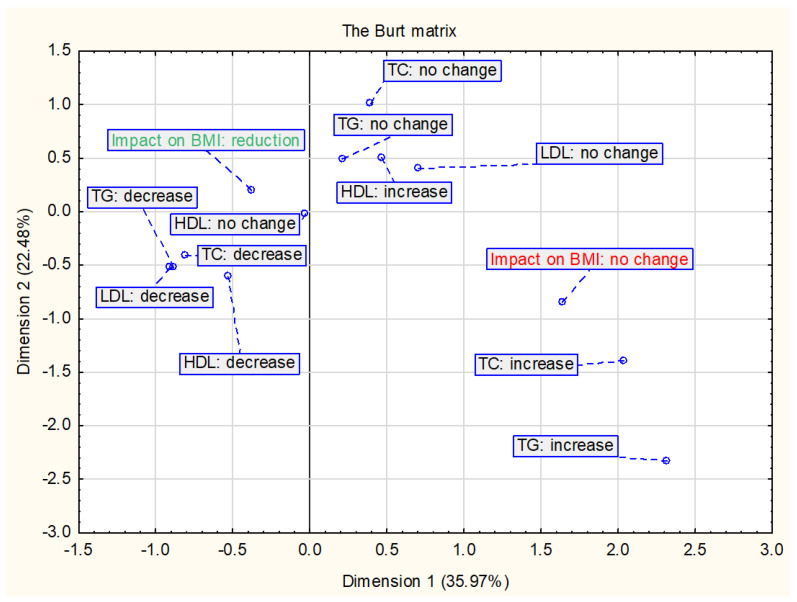
Presentation of the correspondence analysis results defining the relationship between the changes in BMI and/or BMI z-score and changes in lipid parameters (*n* = 16).

Figure 3 shows the results of the relationship between the change in BMI and/or BMI z-score and changes in lipid parameters. The analysis included 16 interventions—studies without all data on lipid parameters were not included. The data indicate that a decrease in BMI is mostly associated with decreases in TC, TG and LDL-C.

**Figure 4:**
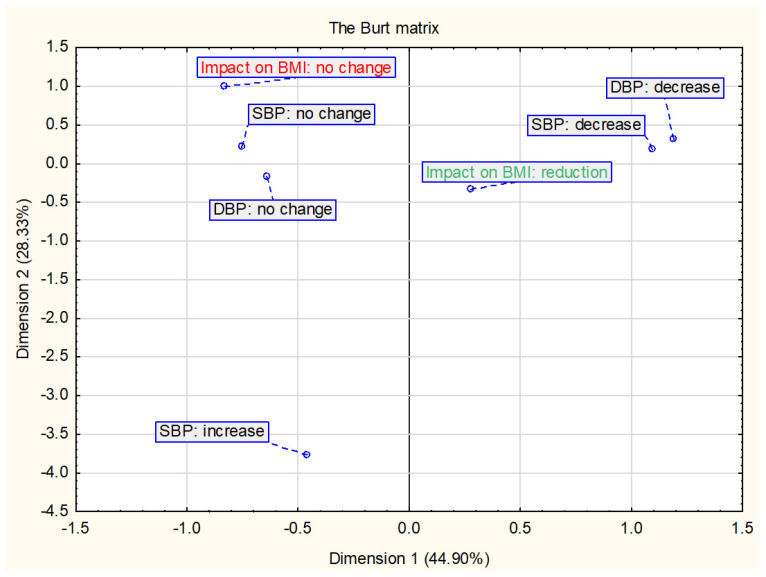
Presentation of the correspondence analysis results defining the relationship between the changes in BMI and/or BMI z-score and changes in blood pressure (*n* = 20).

Figure 4 shows the results of the relationship between the change in BMI and/or BMI z-score and changes in blood pressure. The analysis included 20 in which both SBP and DBP were assessed. The decrease in BMI is most closely related to the decrease in DBP and SBP and no change in BMI with no change in SBP and DBP.

### 2.3. Parental and Therapeutic tEam Involvement

#### 2.3.1. Parental Involvement

In 13 interventions, parents were involved in their children’s obesity treatment program [36,38,42,43,45,46,47,49,51,52]. Nine interventions focused on health promoting lessons and healthy cooking at home [36,38,42,45,46,47,51,52]. In two cases, the parents followed the same diet as the child [49]. In 5 interventions, parents were given advice on how to support their child and encourage them to change unhealthy eating habits and increase physical activity [36,38,45,51,52]. No information about parental involvement was reported in 10 interventions [35,37,39,40,41,44,48,50].

#### 2.3.2. Therapeutic Team Involvement

In the presented studies, most often a dietician or nutrition specialist was included in the therapeutic team (15 interventions) [38,40,41,42,43,44,45,46,47,50,51,52]. Next a physical activity specialist (11 interventions) [35,36,38,40,44,45,46,50,51,52] and a physician (10 interventions) [35,41,42,44,45,46,47,52]. The therapeutic team involved at least two different specialists in 16 interventions [35,36,38,40,41,42,44,45,46,47,50,51,52]. No information about therapeutic team was reported in 5 interventions [37,39,48,49]. Only in two interventions a psychologist was included in the therapeutic team [46,51].

#### 2.3.3. The Impact of Parental and Therapeutic Team Involvement on the BMI and/or BMI z-Score

From the interventions where parents were involved in their children’s obesity treatment program, 10 reported a decrease in BMI and/or BMI z-score [38,42,43,45,46,47,49,51,52]. By comparison, this effect was reported in 8 interventions where parents were not directly involved in the children’s weight loss program [35,37,39,40,41,44,48].

Among the interventions showing a decrease in BMI and/or BMI z-score, in 13 of them, the dietician or nutrition specialist was a member of the therapeutic team [38,40,41,42,43,44,45,46,47,51,52] and the physician in 9 of these interventions [35,41,42,44,45,46,47,52]. A therapeutic team consisting of at least two different specialists participated in 12 out of 18 interventions effective in reducing BMI and/or BMI z-score [35,38,40,41,42,44,45,46,47,51,52], while 2 interventions was supervised only by a dietician or nutrition specialist [43].

**Figure 5:**
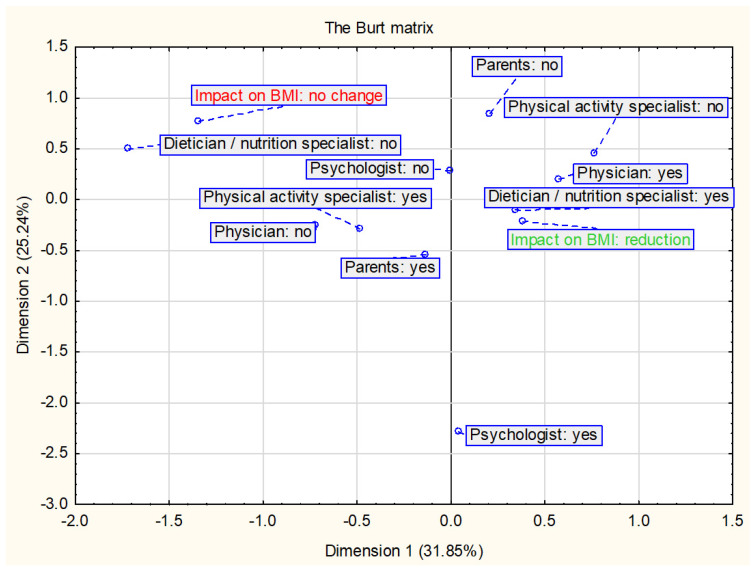
Presentation of the correspondence analysis results defining the relationship between the changes in BMI and/or BMI z-score and parental and therapeutic team involvement (*n* = 18).

The results of the relationships between the change in BMI and/or BMI z-score and parental and therapeutic team involvement are presented in Figure 5. The analysis included 18 interventions in which the participation of parents and at least one specialist in the therapeutic team was indicated. BMI reduction is mostly associated with the presence of a dietician or nutrition specialist and physician in the therapeutic team, then with the participation of the parent. The data analysis also shows an inverse relationship—the lack of participation of a dietician or nutrition specialist in the intervention is related to no changes in the BMI and/or BMI z-score.

#### 2.3.4. The Impact of Parental and Therapeutic Team Involvement on Selected Cardiometabolic Parameters

Parents were involved in 7 interventions which showed an effect on the improvement of at least one lipid parameter [36,42,45,46,49,51,52], while in the remaining 7 parents were absent [35,37,39,40,44,48,50]. In 8 interventions with the participation of a dietician, the effect of the intervention on the improvement of at least one parameter of the lipid profile was demonstrated [40,42,44,45,46,50,51,52], but in another 7 studies the lipid parameters did not change significantly [38,41,43,44,47]. Nine of the 11 interventions in which the physical activity specialist participated showed an improvement in at least one parameter of the lipid profile. [35,36,40,44,45,46,50,51,52].

Of the 9 studies that found a significant reduction in SBP, parents were involved in 6 of the interventions [36,38,43,46,49]. In 6 out of 11 interventions that did not significantly affect the SBP, there was no parental involvement [41,44,48,50]. Similarly, in the case of DBP—no significant changes were noted in 7 out of 13 interventions with parents [36,42,46,47,49,52], and a decrease in 4 out of 10 interventions without parental involvement [37,39,40,44].

From the interventions where a dietician or nutrition specialist was part of the treatment team, a significant reduction in SBP was noted in 5 [38,40,43,46] and DBP in 4 interventions [40,43,44]. For comparison, in 7 interventions with these specialists, no significant change in SBP and DBP was found [41,42,44,47,50,52]. Similarly, in the case of a physician. In 7 and 8 interventions with this participation, no significant changes were found in SBP [41,42,44,47,52] and DBP [35,41,42,44,46,47,52], respectively. Of the interventions with the participation of a physical activity specialist, 4 reported a reduction in SBP [36,38,40,46], but the same number of interventions had no effect on SPB [36,44,50,52]. Similarly, in the case of effects on DBP—5 interventions with a physical activity specialist did not affect DBP [35,36,46,50,52], and only 3 had a significant reduction in DBP [36,40,44]. On the other hand, in 5 out of 7 interventions in which a physical activity specialist did not participate, there was no significant change in both SBP and DBP [41,42,44,47].

### 2.4. Duration of the Intervention

The duration of individual interventions varied—the shortest lasted 4 weeks and the longest 2 years. For the purposes of data analysis, two categories were distinguished because it was hypothesized that longer intervention time is associated with a decrease in BMI. Nine interventions lasted less than 6 months [37,39,44,46,48,49,50], and in the remaining 14—at least 6 months [35,36,38,40,41,42,43,45,47,51,52].

#### The Impact of Duration of the Intervention on the BMI and/or BMI z-Score and Selected Cardiometabolic Parameters

Reduction in BMI and/or BMI z-score was noted in 12 interventions lasting at least 6 months [35,38,40,41,42,43,45,47,51,52] and in 6 interventions lasting less than 6 months [37,39,44,46,48,49]. Of the studies that showed the effect of an intervention on the improvement of at least one lipid parameter, half lasted less than 6 months [37,39,44,46,48,49,50], and half lasted at least 6 months [35,36,40,42,45,51,52]. Similar relations were observed for the influence of the intervention on the values of SBP and DBP.

**Figure 6:**
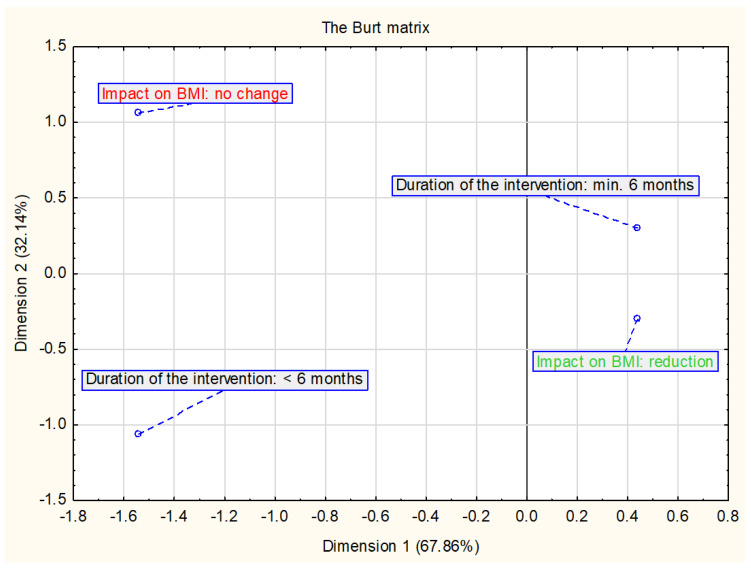
Presentation of the correspondence analysis results defining the relationship between the changes in BMI and/or BMI z-score and duration of the intervention (*n* = 23).

Figure 6 shows the results of the relationship between the change in BMI and/or BMI z-score and the duration of the intervention. The analysis included all interventions assessed in this study (*n* = 23). Data suggest that longer duration of intervention is more associated with a decrease in BMI.

## 3. Discussion

Treatment of obesity in children and adolescents is difficult and can be done in different ways. The most common methods are dietary change (e.g., following a calorie-specific diet), behavioral change (e.g., nutritional education, self-monitoring, setting achievable nutritional goals) and implementing physical activity. The duration of the intervention and the involvement of parents and therapeutic team can also be important to success.

The review summarized interventions aimed at children and adolescents with overweight or obesity, where one of the key factors was changing the diet or eating habits. The use of a diet with a specific caloric value (with or without a caloric deficit) seems to be an effective element of intervention programs in the context of reducing the BMI index and/or BMI z-score. This may also be related with an involvement of a dietician or nutrition specialist in the therapeutic team. In a meta-analysis of 33 studies, researchers in Australia found that the most common interventions in the treatment of obesity in children and adolescents were calorie-restricted diets and the modified Stop/Traffic Light approach. Both of that were effective in reducing body weight [54]. Educational interventions, including nutrition, were also effective in reducing BMI and diastolic blood pressure in children 6–12 years of age for a minimum duration of 6 months [55]. Regarding the duration of the intervention, this study showed an association between an intervention of at least 6 months and a decrease in BMI and/or BMI z-score. Additionally, Janicke et al. [56] showed that longer intervention times are associated with better weight loss outcomes. However, the authors do not indicate the limit of the intervention duration at which better results can be achieved. It is also worth noting that the intensity of intervention programs affects their effectiveness. This is demonstrated by the analysis of data from 42 studies on lifestyle interventions to reduce excess weight [57]. Interventions of 6–12 months have been shown to be effective in losing weight in children and adolescents at intensity levels above the estimated threshold of 26 h of contact, based on the number of designed therapy sessions and the length of each session. The effects of body weight reduction turned out to be better with more estimated contact hours. The best effects were indicated in the case of interventions for which a minimum of 52 contact hours was specified, including a decrease in BMI by 0.22–0.34 and a decrease in blood pressure by 4 mm Hg.

Usually, it is not easy to clearly assess the effectiveness of the actions, because of the complexity of intervention programs and the interaction of various elements. Nutritional interventions are rarely used alone in the treatment of overweight in children and adolescents. Most often they are one of the elements of the intervention program. Similarly, in this analysis, most of the interventions were interdisciplinary, and in addition to nutrition, physical activity was the most common. It was noticed that physical activity was part of 83% of the interventions in which the BMI and/or BMI z-score decreased significantly. These data suggest that multi-component obesity treatment programs in children and adolescents may be an effective tool for short-term weight control. Similar conclusions about the impact of multi-component interventions on weight loss were made by Mead et al. [58] in a review of studies involving children aged 6–11 years and Rajjo et al. [59] in a review of 133 randomized trials. Ho et al. [54] showed that lifestyle interventions reduced BMI by an average of 1.25 kg/m^2^ (BMI z-score: −0.1), and a significant reduction in LDL-C and TG levels and blood pressure in children and adolescents with overweight or obesity aged ≤18 years. Similarly, an analysis of data from 71 studies assessing the effect of weight reduction on the parameters of the lipid profile and blood pressure showed that a decrease in BMI z-score by > 1.2 is a likely factor in reducing the level of LDL-C and by > 0.7 for TG and by > 1 for SBP [60]. Other researchers indicate a reduction in cardiometabolic risk with a decrease in BMI z-score by ≥ 0.25 in adolescents with obesity, and greater health benefits can be achieved with a decrease in BMI z-score by a minimum of 0.5 [61]. However, the authors of the studies emphasize that there is a need for further research in this area, necessary to determine the optimal duration and intensity of intervention.

Many researchers indicate that the family plays a key role in the effectiveness of overweight treatment programs in children and adolescents [62,63]. Parental involvement is intended to support the child in making correct food choices and health behaviors. Moreover, parents are role models for children, especially in the younger age groups where incorrect parental attitudes and behavior seem to make these children overweight [53,64,65]. The studies included in this review did not clearly show the impact of parental involvement in the childhood obesity treatment program on the reduction of BMI and/or BMI z-score. Parental involvement in the intervention was also less associated with a decrease in BMI compared to a dietician or nutritional specialist and physician, but greater than that of a psychologist. This may result from the large age diversity of the participants of the analyzed studies. Adolescents may have greater trust in specialists, and younger children in their parents. Researchers indicate that better effects of interventions involving parents are achieved by children under 12 years of age [54]. On the other hand, the intervention in the treatment of childhood obesity, but addressed only to parents, has been shown to be effective in children aged 5–11 [66]. In the case of obesity prevention strategies, it is pointed out that they should be implemented in preschool children because of the greater chance of success [65]. In the systematic review, Kelishadi and Azizi-Soleiman [64] showed that parental participation in childhood obesity treatment programs was an important element in children’s success. However, the authors indicate that parents with low self-confidence were more likely to quit the program. Such observations may indicate the need to assess the willingness of parents to change before starting the program [53,64]. Another solution may be to find methods to increase parents’ motivation to participate in the intervention. Ho et al. [54] showed that an additional factor influencing the effectiveness of family interventions was encouraging parents to lose weight if they were overweight or offering them a free swimming pass.

### Strengths and Limitations

In the literature, there is still insufficient data indicating what factors of lifestyle intervention programs in children and adolescents with excess body weight contribute to success in the context of weight loss and improvement of selected cardiometabolic parameters. The strength of this review is the identification of several components of the intervention that are most closely related to the effect on BMI, and therefore on selected cardiometabolic factors. The BMI and/or BMI z-score index, which are commonly used in studies, was used to assess the change in body weight. Another advantage of the review is the inclusion of studies that assessed selected cardiometabolic factors, although not all of the assessed parameters were distinguished in each study. This review analyzed only the occurrence of the changes in all parameters after interventions, not their magnitude. However, the study has some limitations. This includes a large differentiation of the analyzed interventions due to the type of research or the different size of the respondent groups. The studies used different methods of intervention. The heterogeneity of tools used for assessment of physical activity level or nutritional adaptation programs generate problems in the evaluation of the effectiveness of these components in different interventions. Standardized methods and objective tools for the assessment of the physical activity level and nutritional adaptation should be established. However, it was possible to define similar concepts of intervention, such as diet, behavioral methods and physical activity. The analyzed interventions were conducted by various therapeutic teams. Intervention time covers only the generally expressed time of intervention without specifying the exact number of hours and frequency of meetings with the therapeutic team. It is also challenging to target programs at different age groups, for example due to the different potential influence of parents on adolescents and children. Nevertheless, it was possible to distinguish common features of the intervention. The aim of this study was to update the state of the knowledge from the last decade. This approach may limit the strength of some conclusions. In clinical practice, the method of intervention should always depend on the patient’s health status, the presence of comorbidities or the medications taken. There is a need for further analysis with regard to other obesity related disorders, including disorders of glucose metabolism.

## 4. Conclusions

The analyzed studies suggest that in the treatment of childhood obesity, interventions related to changes in lifestyle including diet and physical activity, participation of a dietician or nutritional specialist and physician in a therapeutic team, and longer duration of intervention, are effective. The data indicate that a decrease in BMI is most associated with decreases in total cholesterol, triglycerides, low density lipoprotein cholesterol, diastolic and systolic blood pressure. On the other hand, no change in BMI and/or BMI z-score in children and adolescents with overweight or obesity seems to be related to an increase in total cholesterol and triglycerides, and no change in blood pressure. However, further research is needed to identify the most effective lifestyle intervention model in treating excess body weight and in improving cardiometabolic parameters in children and adolescents. There is also a need for further research related to the maintenance of weight loss among overweight and obese children as well as adolescents participating in intervention programs.

## Figures and Tables

**Table 2:** Characteristics of the lifestyle intervention studies in children and adolescents with overweight or obesity.

Country, Year [Reference]	Participants, Criteria for Overweight and Obesity	Duration of the Intervention, Study Design	Therapeutic Team	Characteristics of the Intervention	Control Group	Effect of the Intervention
Belgium, 2015 [35]	*n* = 33, 27.3% boys, 12–18 years (mean 15.4 ± 1.5 years), BMI ≥ 97th pc (< 16 years old), BMI ≥ 35 (> 16 years old)	10 months, quasi-randomized trial	pediatrician, physiotherapists	- nutritional intervention: low calorie diet (1500–1800 kcal a day); - physical activity: daily 2-h supervised games and lifestyle activities, 2 h physical education a week at school, 3 supervised training sessions every week;	*n* = 28, 21.4% boys, years (mean 15.1 ± 1.2 years): - usual medical care focused on reducing calories and encouraging sports;	Compared to control group: ↓ % BF, BMI, BMI z-score, BW, LDL-C ↑ HDL-C, SBP ↔ DBP, TC
China, 2015 [36]	*n* = 90, 38.9% boys, 7–12 years (mean 9.41 ± 1.03 years), BMI criteria according to WGOC	1 year, nonrandomized controlled trial with cluster sampling	medical research postgraduate students, physical trainer	Comprehensive intervention group: - nutritional intervention: individual dietary behavior goal settings, dietary monitoring; - physical activity: recommendations of 60 min physical activity daily, individual goal settings to improve physical activity levels, monitoring using accelerometers; - parental involvement: nutrition and physical activity education, encouraging children to modify unhealthy eating behavior and increase physical activity; - rewards for achieving goals;	*n* = 136, 32.4% boys, 7–12 years, mean age 9.16 ± 1.12 years: - no intervention;	Compared to diet only intervention group: ↓ % BF, BMI, HDL-C, SBP ↔ DBP, LDL-C, WC ↑ TC, TG Compared to control group: ↓ % BF, DBP, SBP ↔ BMI, HDL-C, LDL-C, WC ↑ TC, TG
	*n* = 96, 42.7% boys, 7–12 years (mean 9.27 ± 1.34 years), BMI criteria according to WGOC			Diet only intervention group: - nutritional intervention: nutritional education, textbooks for nutrition education in the form of cartoons; - parental involvement: nutrition education;		Compared to control group: ↔ BMI, DBP, LDL-C, SBP, TG, WC ↑ % BF, HDL-C, TC
China, 2014 [37]	*n* = 20, 55% boys, 7–17 years, BMI ≥ 95th pc	4 weeks, intervention study	no data	- nutritional intervention: energy restricted diet ranging from 1338 to 1883 kcal a day depending on age (19.4 ± 2.4% energy from protein, 2.7 ± 3.7% from fat and 60.0 ± 4.4% from carbohydrates); - physical activity: four supervised 45-min physical activity sessions;	no control group	Compared to baseline: ↓ BFM, BMI, BW, DBP, LDL-C, SBP, TC, TG, WC, WHR ↔HDL-C
Denmark, 2016 [38]	*n* = 55, 47.3% boys, 11–13 years (mean 12.0 ± 0.4 years), BMI criteria according to IOTF	1 year, randomized controlled trial	dietician, trained instructors, school nurses	Day-camp intervention (6 weeks) and family-based intervention (after day-camp): - nutritional intervention: day-camp meals prepared according to the national Danish dietary recommendations (no caloric restriction), dietary course; - physical activity: minimum 3 h of exercise a day at day-camp, “activity day” during family-based intervention; - parental involvement: dietary course, information about healthy cooking, advice on how best to support the child’s health behavior;	Standard intervention arm (SIA—6 weeks) (*n* = 52, 41.2% boys, 11–13 years (mean 12.0 ± 0.4 years): - 2-h weekly exercise session; - a single health and lifestyle educational session for the parents;	After 6 weeks, compared to SIA group: ↓ % BF, BMI, BMI z-score, TC/HDL-C ratio, SBP, WC, % of abdominal fat, clustered cardiovascular risk z-score ↔ FFM, TG After 52 weeks, compared to SIA group: ↓ BMI, BMI z-score, TC/HDL-C ratio, clustered cardiovascular risk z-score ↔ % BF, FFM, SBP, TG, WC, % of abdominal fat
Denmark, 2012 [39]	*n* = 117, 43.6% boys, mean age 12.1 ± 1.3 years, no criteria for assessing obesity	10 weeks, intervention study	no data	- nutritional intervention: weight loss camp, approximate daily energy consumption was 1547 kcal/6475 kJ (60% energy from carbohydrates, 16% energy of protein and 24% energy from fat), 3 healthy meals a day at set times plus 3 healthy snacks, - physical activity: at least 1 h a day individual or group activity;	no control group	Compared to baseline: ↓ % BF, BFM, BMI z-score, BW, DBP, LDL-C, SBP, TC, TG, WC, WHR ↔ HDL-C 12-month follow-up compared to the end of the weight loss camp: ↑ BFM, BMI z-score, BW, DBP, LDL-C, SBP, TG, TC, WC, WHR ↔ % BF, HDL-C 12-month follow-up compared to baseline: ↓ % BF, BFM, BMI z-score ↑ BW, DBP, SBP ↔ HDL-C, LDL-C, TC, TG, WC, WHR
France, 2013 [40]	*n* = 28, 32% boys, mean age 14.2 ± 1.5 years, BMI > 97th pc and BMI z-score > 3	9 months, intervention study	dieticians, fitness teacher	- nutritional intervention: balanced diet 2300–2500 kcal (30% energy from fat, 14% energy from proteins, 56% energy from carbohydrates), nutritional education; - physical activity: 45–60 min exercises at least 5 times a week, physical education lesson;	*n* = 20, 40% boys, mean age 14.9 ± 1.6 years: - healthy adolescents; - no intervention;	Compared to baseline: ↓ BFM, BMI, BMI z-score, BW, DBP, SBP, WC ↑ HDL-C ↔ LDL-C, TC, TG Compared to control group: ↑ BMI, BMI z-score, BW ↔ DBP, SBP
Greece, 2012 [41]	*n* = 21, 47.6% boys, 8–18 years (mean 12.8 ± 2.1 years), BMI > 95th pc	6 months, intervention study	dietician, pediatric endocrinologist	Ketogenic diet group: - nutritional intervention: information about the selection of products and composing a ketogenic diet, no caloric restriction, individual dietary counselling (education and counselling on the diet); - physical activity: encouraging a minimum of 1 h of vigorous physical activity a day;	no control group	Compared to baseline: ↓ BFM, BMI, BW, WC ↔ DBP, HDL-C, LDL-C, SBP, TC, TG Compared to hypocaloric diet group: ↔ BFM, BMI, BW, DBP, HDL-C, LDL-C, SBP, TC, TG, WC
	*n* = 17, 41.2% boys, 8–18 years (mean 12.7 ± 2.8 years), BMI > 95th pc			Hypocaloric diet group: - nutritional intervention: low calorie diet (-500 kcal compared to individual daily energy requirements; 28–33% energy from fat and 50–55% from carbohydrates), individual dietary counselling (education and counselling on the diet); - physical activity: encouraging a minimum of 1 h of vigorous physical activity a day;		Compared to baseline: ↓ BFM, BMI, BW, WC ↔ DBP, HDL-C, LDL-C, SBP, TC, TG
Italy, 2015 [42]	*n* = 90, 49% boys, ≥ 6 years (mean 9.7 ± 2.6 years), no criteria for assessing obesity	1 year, intervention study	dietician, pediatrician	- nutritional intervention: normocaloric diet by age and sex (12–15% energy from protein, 25–30% from fat and 55–60% from carbohydrates), nutritional education; - physical activity: at least 60 min a day, physical activity education; - parental involvement: nutrition guidelines, telephone contact with a dietician and pediatrician;	no control group	Compared to baseline: ↓ BMI z-score, TG, TG/HDL-C ratio ↑ HDL-C ↔ DBP, LDL-C, LDL-C/HDL-C ratio, SBP, TC, TC/HDL-C ratio, WC
Italy, 2012 [43]	*n* = 11, mean age 118.0 ± 19.6 months, BMI z-score ≥ 2	6 months, randomized controlled trial	dietician	Low Glycemic Index diet: - nutritional intervention: a hypocaloric diet that provided an energy intake 30% less than the intake sufficient to maintain the ideal body weight (15–20% energy from protein, 25–30% from fat and 50–60% from carbohydrates, glycemic index = 60), individual dietary counselling, 7-day dietary records; - parental involvement: individual dietary counselling;	no control group	Compared to baseline: ↓ BMI, BMI z-score, DBP, SBP, WC ↔ HDL-C, TC, TG Compared to HGI group: ↓ BMI, BMI z-score, TG ↔ HDL-C, TC
	*n* = 11, mean age 113.9 ± 19.4 months, BMI z-score ≥ 2			High Glycemic Index diet group (HGI): - nutritional intervention: a hypocaloric diet that provided an energy intake 30% less than the intake sufficient to maintain the ideal body weight (15–20% energy from protein, 25–30% from fat and 50–60% from carbohydrates, glycemic index = 90), individual dietary counselling, 7-day dietary records; - parental involvement: individual dietary counselling;		Compared to baseline: ↓ BMI, BMI z-score, DBP, SBP ↔ HDL-C, TC, TG, WC
Korea, 2019 [44]	*n* = 44, 63.6% boys, 6–16 years (mean 12.1 ± 2.2 years), BMI > 85th pc	16 weeks, intervention study	doctor, clinical dietician, social workers, nurses	Usual care group: - nutritional intervention: one-to-one nutritional counselling and education, - physical activity: goal settings—minimum 8000 steps a day and reduce inactivity time, physical activity tracker;	no control group	Compared to baseline: ↑ BFM, LM ↔ % BF, BMI z-score, DBP, HDL-C, LDL-C, SBP, TG, WC
	*n* = 26, 65.4% boys 6–16 years (mean 12.8 ± 1.7 years), BMI > 85th pc		doctor, clinical dietician, exercise specialists, social workers, nurses	Exercise group: - nutritional intervention: one-to-one nutritional counselling and education, - physical activity: exercise for 60 min at 3 days a week, physical activity tracker and the daily exercise journal;		Compared to baseline: ↑ LM ↓ % BF, BMI z-score, DBP, LDL-C ↔ BFM, HDL-C, SBP, TG, WC Compared to usual care group: ↓ BMI z-score
Norway, 2011 [45]	*n* = 230, 47.4% boys, 7–17 years, body weight > 97.5 pc for height	1 year, intervention study	clinical nutritionist, pediatrician, physical activity specialist, public health nurses	- nutritional intervention: nutritional education, dietary counselling; - physical activity: encouraging to exercise at least 60 min a day and to limit the time spent watching television and computer activities; - parental involvement: dietary and medical counselling;	no control group	Compared to baseline: ↓ BMI z-score, LDL-C, TC ↔ HDL-C, TG
Switzerland, 2011 [46]	*n* = 203, 56.2% boys, mean age 14.1 ± 2.0 years, BMI > 98th pc	2 months, prospective study	dietician, exercise therapist pediatrician, psychologist, nurses	- nutritional intervention: a balanced diet with restricted energy value of 1200–1600 kcal a day based on basal body weight (15–20% energy from protein, 25–30% from fat and 55–60% from carbohydrates), nutritional education, individual consultations, cooking classes; - physical activity: 60–90-min group exercise sessions twice daily, 4–5 h exercise session once a week and at least 60-min a day supervised ergometric cycling on weekends; - parental involvement: theoretical and practical counselling; - behavior modifications: contracting in emergency situations self-control of calorie intake and body weight, praise and stimulus control, increasing self-esteem, responsibility and problem-solving strategies, relaxation techniques; - 6- and 12-months follow up;	no control group	Compared to baseline: ↓ % BF, BFM, BMI z-score, BW, HDL-C, LDL-C, SBP, TC, TG ↔ DBP 6- and 12-months follow-up compared to baseline: ↓ % BF, BFM, BMI z-score, BW ↑ LM
Thailand, 2015 [47]	*n* = 25, 64% boys, 9–16 years (mean 11.9 ± 1.9 years), BMI criteria according to IOTF	6 months, prospective randomized controlled trial	dietician, pediatrician	- nutritional intervention: low-glycemic index diet, 1400–1500 kcal a day (15–20% energy from protein, 30–35% from fat and 50–55% from carbohydrates), nutritional education, 3-day dietary records; - physical activity: 30 min a day at least three times a week, reducing sedentary activity, physical activity questionnaire; - parental involvement: nutritional education;	*n* = 27, 70.3% boys, 9–16 years, mean age 12.0 ± 2.1 years: - 1200–1300 kcal a day, low-fat (25% energy from fat) high fiber diet; - physical activity the same as in the intervention group;	Compared to baseline: ↓ BMI z-score ↔ % BF, DBP, HDL-C, LDL-C, SBP, TC, TG, WC Compared to control group: ↔ % BF, BMI, BMI z-score, HDL-C, LDL-C, TC, TG, WC
United States, 2015 [48]	*n* = 12, 8.3% boys, 13–17 years (mean 15.2 ± 1.3 years), BMI > 95th pc	14–18 weeks, intervention study	no data	- nutritional intervention: energy value of the diet based on basal body weight (1400, 1600 or 1800 kcal a day; 20–25% energy from protein, 15–20% from fat and 45–55% from carbohydrates, nutrition courses; - physical activity: physical training 3 times a week; - behavioral counselling;	no control group	Compared to baseline: ↓ BMI, BMI z-score, BW, LDL-C, TC, WC ↔ DBP, SBP, TG ↑ HDL-C
United States, 2015 [49]	*n* =14, 36% boys, 9–18 years, BMI ≥ 95th pc	4 weeks, prospective randomized trial	no data	Plant-based no added fat diet group: - nutritional intervention: instructions to avoiding all animal products and fat, reducing the consumption of nuts and avocados, nutritional and cooking education, 3-day diet diary during the study; - parental involvement: the same diet follow by one parent; - a scholarship of fifty dollars a week;	no control group	Compared to baseline: ↓ BMI z-score, LDL-C, SBP, TC ↔ BW, DBP, HDL-C, TG, WC Compared to AHA group: ↓ BMI z-score ↔ BW, DBP, HDL-C, LDL-C, SBP, TC, TG, WC
	*n* = 14, 36% boys, 9–18 years, BMI ≥ 95th pc			The American Heart Association diet group (AHA): - nutritional intervention: diet based on AHA guidelines (30% energy from fat, 7% energy from saturated fat, less than 300 mg cholesterol, less than 1500 mg of sodium daily), nutritional and cooking education, 3-day diet diary during the study; - parental involvement: the same diet follow by one parent; - a scholarship of fifty dollars a week;		Compared to baseline: ↓ HDL-C, WC ↔ BMI z-score, BW, DBP, LDL-C, SBP, TC, TG
United States, 2015 [50]	*n* = 20, 55% boys, 10–19 years (mean 14.3 ± 2.1 years), BMI > 85th pc	12 weeks, intervention study	nutritionist, trainer	- nutritional intervention: dietary goals based on DASH (Dietary Approaches to Stop Hypertension) diet guidelines, weekly conversations with nutritionist, food recording; - physical activity: training sessions 3 times a week; - $15 gift card every 2 weeks;	no control group	Compared to baseline: ↓ LDL-C, TC, WHR ↔ BMI, BMI z-score, BW, DBP, HDL-C, SBP, TG
United States, 2011 [51]	*n* = 186, 33.9% boys, 11–18 years (mean 13.7 ± 1.8 years), BMI ≥ 95th pc	6 months, intervention study	dietician, behavioral support specialist, exercise physiologist	- nutritional intervention: individual dietary counselling, nutritional education, individual dietary goals, self-monitoring of progress, grocery store tour with parents and a nutritionist; - physical activity: 3 exercise sessions a week, self-monitoring of progress; - parental involvement: individual dietary counselling, nutritional education, meetings with a behavioral support specialist, grocery store tour with child and a nutritionist; - meetings with a behavioral support specialist, two $100 gift cards for the grocery store;	no control group	Compared to baseline: ↓ % BF, BMI, BMI z-score, HDL-C, LDL-C, TC, TG
United States, 2011 [52]	*n* = 105, 44.8% boys, 8–16 years (mean 12.0 ± 2.5 years), BMI ≥ 95th pc	2 years, randomized controlled trial	dieticians, physicians, exercise physiologists, social worker	- nutritional intervention: nutritional education to modify eating behavior; - physical activity: 50 min of exercises at 2 times a week, encouraging to exercise for an additional 3 days a week at home and reducing a sedentary lifestyle; - parental involvement: nutritional education, highlighting the role of the parent in modelling the change in healthy behavior; - behavior modifications: techniques for self-awareness, goal setting, stimulus control, training, coping and cognitive skills behavior strategies; - 12-month follow up—no active intervention, encouragement to remain active and to use acquired nutritional knowledge;	*n* = 69, 31.9% boys, 8–16 years (mean 12.5 ± 2.3 years): - general diet and exercise counselling every 6 months.	Compared to control group—end of intervention: ↓ % BF, BFM, BMI, BMI z-score, BW, TC ↔ DBP, HDL-C, LDL-C, SBP, TG Compared to control group after 12-month follow-up: ↓ % BF, BFM, BMI, BMI z-score, BW, LDL-C, TC ↔ DBP, HDL-C, SBP, TG

↓—decrease; ↑—increase; ↔—no change; BF—body fat; BFM—body fat mass; BMI—body mass index; BW—body weight; DBP—diastolic blood pressure; FFM—free fat mass; HDL-C—high-density lipoprotein cholesterol; IOTF—International Obesity Task Force; LDL-C—low-density lipoprotein cholesterol; LM—lean mass; pc—percentile; SBP—systolic blood pressure; TC—total cholesterol; TG—triglycerides; WC—waist circumference; WGOC—Working Group for Obesity in China; WHR—waist to hip ratio.

## Data Availability

Data sharing is not applicable to this article.
